# An integrated EEG and eye-tracking approach for the study of responding and initiating joint attention in Autism Spectrum Disorders

**DOI:** 10.1038/s41598-017-13053-4

**Published:** 2017-10-19

**Authors:** Lucia Billeci, Antonio Narzisi, Alessandro Tonacci, Beatrice Sbriscia-Fioretti, Luca Serasini, Francesca Fulceri, Fabio Apicella, Federico Sicca, Sara Calderoni, Filippo Muratori

**Affiliations:** 1 Institute of Clinical Physiology, National Research Council of Italy, Via Moruzzi 1, 56124 Pisa, Italy; 2 IRCCS Stella Maris Foundation, Viale del Tirreno 331, 56018 Calambrone, (PI) Italy; 3 Azienda Ospedaliero-Universitaria di Parma, Strada Abbeveratoia, 2, 43100 Parma, Italy; 4 Department of Clinical and Experimental Medicine, University of Pisa, via Savi 10, 56126 Pisa, Italy

## Abstract

Autism Spectrum Disorders (ASD) are characterised by impairment in joint attention (JA), which has two components: the response to JA and the initiation of JA. Literature suggests a correlation between JA and neural circuitries, although this link is still largely unexplored in ASD. In this pilot study, we aimed at investigating the neural correlates of responding and initiating JA in high-functioning children with ASD and evaluating the changes in brain function and visual pattern after six months of rehabilitative treatment using an integrated EEG/eye-tracking system. Our results showed that initiating and responding JA subtend both overlapping (i.e. frontal and temporal) and specialized (i.e. parietal for responding JA and occipital for initiating JA) neural circuitries. In addition, in a subgroup of subjects, we observed trends of changes in both brain activity and connectivity after rehabilitative treatment in both the two tasks, which were correlated with modifications in gaze measures. These preliminary results, if confirmed in a larger sample, suggest the feasibility of using the proposed multimodal approach to characterise JA-related brain circuitries and visual pattern in ASD individuals and to monitor longitudinal changes in response to rehabilitative intervention.

## Introduction

Joint attention (JA) is described as the ability to coordinate visual attention with another person and then shift the gaze toward a shared object or event^1^. Deficits in JA are known to be one of the prominent features of Autism Spectrum Disorders (ASD)^2–4^, and have been correlated not only with social and communicative problems, but also with deficits in language development^5^. Children who will later receive a diagnosis of ASD show by 6 months of age a reduced ability to pay attention to social cues^6^,^7^. In addition, face processing deficits seem to represent a sensitive marker for the detection of ASD risk in toddlers and siblings^8–10^.

Literature reports on two main components of JA: the response to JA and the initiation of JA. Responding to JA (RJA) was described as the ability to shift visual attention following other’s social cues such as gaze or pointing; initiating JA (IJA) was reported as the ability to seek other’s attention using one’s own direction of gaze or gestures with the aim of sharing an experience. IJA and RJA are thought to be as two interrelated aspects of JA linked to different processes during infant development^2^. Several studies have analysed in depth joint behaviours^11–14^, most of which having focused on the responding abilities^12,15^, that precede the initiation skills during typical development.

Eye-tracking has recently been used during JA tasks in ASD, focusing in particular on RJA and gaze accuracy. For example, Chawarska and colleagues^16^, using images of faces appropriately processed to reproduce the effect of gaze shift, did not find any differences in gaze accuracy between children with ASD and peers with typical development (TD), but reported that the difficulties in gaze following emerge along with the severity of the socio-communicative impairment. On the other hand, Falck-Ytter and colleagues^17^, using video sequences where a model either looked, or pointed, or looked and pointed toward an object, found that preschoolers with ASD performed fewer and slower correct gaze shifts compared to TD preschoolers. More recently, the same group explored the response to JA in younger children, using videos in which a female model looked and turned her head towards an object, and found no differences between ASD subjects and TD peers. However, the ASD group showed a weaker first fixation preference for the attended object compared to TD, suggesting an initial processing bias for attended objects in ASD individuals^18^. These contradictory results could be ascribed to the different nature of the stimuli used in the tasks (images or videos), and/or to the different social cues (gaze shift, pointing, head movement), and/or, most importantly, to the focus of most studies on the responding component of JA that could be likely only partially impaired in ASD.

Recently, we have described the visual patterns of toddlers with ASD and TD searching for differences in RJA and IJA tasks in an eye-tracking scenario^19^. We found no differences in the RJA task between the two groups, whereas different gaze patterns emerged in the IJA tasks. We have hypothesised that these differences were due to ASD-related impairments in visual disengagement from faces, in global scanning of the scene, and in the ability to anticipate object’s action.

Literature has suggested a correlation between JA abilities and specific neural circuitry. Previous studies indicated a direct relationship between increased frontolimbic neural circuit connectivity at 6 months and subsequent RJA abilities at 9 months in TD individuals^20^.

Some functional magnetic resonance imaging (fMRI) studies also explored the neural correlates of JA in adults with ASD. In a RJA task consisting of watching an animation of a gaze shift towards (congruent condition), or away from (incongruent condition) a flashing target, adults with ASD did not show any modulation in the activation of the superior temporal sulcus (STS) while looking at the congruent vs. incongruent condition like TD adults^21^. Another study has explored the neural substrates of IJA and RJA in adults with ASD in a paradigm using live interactions and revealed both distinct (ventromedial prefrontal cortex for RJA, and intraparietal sulcus and middle frontal gyrus for IJA) and overlapping regions (dorsal medial prefrontal cortex, and right posterior superior temporal sulcus) for RJA and IJA abilities^22^.

Recently, few studies have explored JA in ASD patients using combined eye-tracking and brain-based measures. Specifically, Elison and colleagues^23^ collected both eye-tracking and diffusion tensor imaging data in high-risk positive, high-risk negative and low-risk infants. They reported longer visual orienting latencies in high-risk positive subjects compared to the other groups and detected a correlation between visual orienting and microstructural organization of the splenium of the corpus callosum only in typical infants. Only one recent study^24^ used a combined EEG/eye-tracking paradigm to explore the correlates of RJA in adolescents with ASD, during congruent and incongruent tasks. The authors reported that, across conditions, adolescents with ASD exhibited reduced right hemisphere temporal–central alpha coherence compared to TD controls while no significant between-groups differences in visual fixation were reported.

In broader terms, multimodal neuroimaging and neurophysiological approaches associated with eye-tracking measures seem to be crucial for providing new information on how the structural, functional and behavioural alterations in early attention abilities reported in ASD patients are related.

The aim of this pilot study is to investigate the neurophysiological and gaze correlates of both RJA and IJA tasks in high-functioning children with ASD. To this aim, we developed a new paradigm, based on our previous study in toddlers^19^, and recorded simultaneous EEG/eye-tracking data in an integrated approach. We also aimed at quantifying the longitudinal changes in neurophysiological and gaze correlates of JA after treatment. The work was carried out within the framework of MICHELANGELO, a project funded by the European Commission (FP7-ICT-288241) (http://www.michelangelo-project.eu/).

This study has several elements of novelty, namely: (a) exploring the neural correlates of RJA and IJA in children with ASD; (b) integrating EEG with eye-tracking to explore the visual patterns of the two components of JA (to our knowledge, the only study that used a similar protocol in ASD, focusing only on the RJA, was indeed the study by Jaime and colleagues^24^); and, (c) evaluating the changes in JA-related brain regions and visual patterns in a longitudinal way. This new methodology has been tested in a pilot study in eleven children with ASD, of which a subgroup of seven performed the treatment. Given the high novelty of the approach, we aimed at evaluate the feasibility of the method in this small sample size and we did not presume to test the efficacy of the approach.

Based on previous EEG literature in TD, we expect that both overlapping and different circuitries activate during RJA and IJA tasks. In addition, following our previous study on toddlers^19^, we hypothesise a prevalent impairment in initiating, and expect some longitudinal improvement after 6-month treatment; finally, given that a decreased coherence in ASD patients compared with TD individuals has been reported^24^, we hypothesise an increase of coherence after the 6-month treatment focused on JA.

## Results

### Neural correlates of RJA and IJA tasks at T0

Differences in EEG measures between RJA and IJA task were statistically assessed in the whole group of subjects at T0.

Repeated measures ANOVA for relative power revealed a significant effect of task x band in right frontal (F = 7.32, p = 0.02, η^2^ = . 449), right parietal (F = 10.34, p = 0.01, η^2^ = . 771), left temporal (F = 5.50, p = 0.03, η^2^ = . 408), right occipital (F = 13.93, p = 0.001, η^2^ = . 635) and left occipital (F = 4.18, p = 0.009, η^2^ = 0.374) regions.

Successive univariate analyses were performed to evaluate between-condition differences in power within the five different bands. A series of Bonferroni adjusted paired-sample t-tests resulting in a significance of p < 0.01 were applied. These tests showed that in RJA task right frontal relative power was significantly higher in alpha band (RJA: 6.5 ± 1.19, IJA: 4.3 ± 1.22, p = 0.008, d = 1.00) and marginally significantly higher in theta band (RJA: 13.5 ± 2.49, IJA: 11.25 ± 2.35, p = 0.01, d = 0.84). In addition, relative power was significantly increased in RJA task in right parietal areas in alpha band (RJA: 8.45 ± 2.32, IJA: 6.55 ± 2.17, p = 0.009, d = 0.95); left temporal power was marginally significantly increased in theta band (RJA: 12.3 ± 1.87, IJA: 11.0 ± 1.75, p = 0.01, d = 0.93) and right occipital power was significantly increased in theta band (RJA: 13.09 ± 3.14, IJA: 11.87 ± 3.15, p = 0.003, d = 1.30)

**Figure 1:**
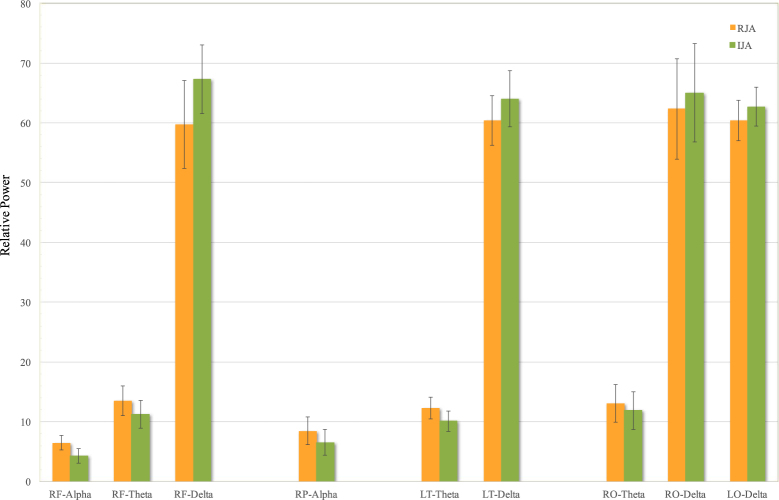
Differences in relative power between responding (RJA) and initiating (IJA) joint attention task at T0 in frontal, parietal, temporal and occipital regions. RF: right frontal, RP: right parietal, LT: left temporal, RO: right occipital LO: left occipital.

Conversely, during IJA there was a marginally significantly higher right frontal power in delta band (RJA: 59.7 ± 7.33, IJA: 67.28 ± 5.75, p = 0.01, d = −0.95) and higher left temporal power in delta band (RJA: 60.4 ± 4.17, IJA: 64.0 ± 4.7, p = 0.01, d = −0.90). Moreover, right occipital power was significantly increased in delta band (RJA: 62.32 ± 8.41, IJA: 65.02 ± 8.28, p = 0.003, d = −1.27) while left occipital power was marginally significantly increased in delta band (RJA: 60.4 ± 3.40, IJA: 62.7 ± 3.22, p = 0.01, d = −0.90). Differences in RJA and in IJA in the various brain regions and frequency bands are summarised in Fig. 1.

With regard to coherence, the ANOVA revealed a significant effect of task x band for parietal (F = 5.89, p = 0.018, η^2^ = 0.890), occipital (F = 3.30, p = 0.02, η^2^ = 0.369) and right fronto-occipital coherence (F = 5.68, p = 0.02, η^2^ = 0.820).

Also in this case, to evaluate between-condition differences in coherence within the five different bands, a series of Bonferroni adjusted paired-sample t-tests resulting in a significance of p < 0.01 were performed. These univariate tests revealed higher inter-hemispheric parietal coherence during RJA task in theta band (RJA: 0.23 ± 0.01, IJA: 0.18 ± 0.01, p = 0.008, d = 1.03).

On the other hand, in the IJA task, there was a higher delta occipital coherence (RJA: 0.42 ± 0.17, IJA: 0.52 ± 0.16, p = 0.003, d = −1.20) and delta right frontal-occipital coherence (RJA: 0.26 ± 0.07, IJA: 0.31 ± 0.07, p = 0.003, d = −1.20).

### Longitudinal changes in eye-tracking measures

Longitudinal changes were qualitatively evaluated in the subgroup of seven subjects who performed the six-month treatment.

Regarding the eye-tracking, we observed a trend of an increase in the cumulative fixation duration for face at T1 compared to T0 for control (T0: 1.15 ± 6.9, T1: 51.5 ± 10.2), RJA (T0: 33.4 ± 9.4, T1: 51.6 ± 12.6) and IJA (T0: 21.6 ± 8.7, T1: 48.2 ± 15.4) tasks. In the RJA task, the cumulative fixation duration for the target object slightly increased, while the cumulative fixation duration for the non-target object remained quite stable.

In the IJA task, children with ASD tended to decrease the cumulative fixation duration for the target object (the moving one) and to increase the cumulative fixation duration for the non-target object.

### Longitudinal changes EEG measures

**Figure 2:**
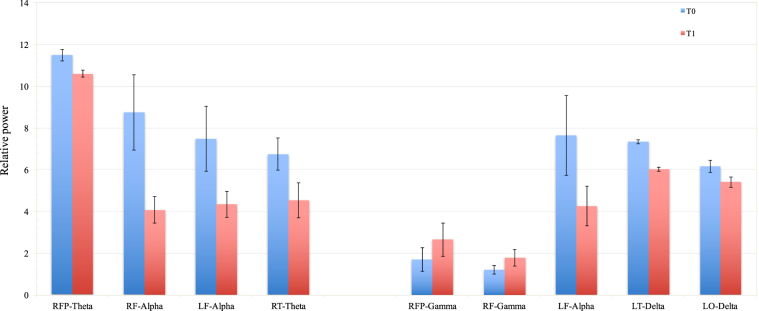
Trends of changes in relative power in responding (RJA) (left side) and initiating (IJA) (right side) joint attention task between T0 and T1. RFP: right frontal polar, LF: left frontal, RF: right frontal, RT: right temporal, LT: left temporal, LO: left occipital.

Some trends were observed in EEG power and coherence between T0 and T1 both in RJA and IJA tasks. As regard the RJA task, we observed a trend of decreasing in relative power in alpha band in right temporal (T0: 6.75 ± 1.56, T1: 4.54 ± 1.67), left frontal (T0: 7.47 ± 2.11, T1: 4.35 ± 1.22) and right frontal areas (T0: 8.74 ± 2.61, T1: 4.08 ± 1.26). A reduction in theta band in the right frontal polar area was also noticed (T0: 11.48 ± 0.54, T1: 10.6 ± 0.34). Figure 2 shows trends of changes in power between T0 and T1.

Looking at coherence, a trend of increasing of the right inter-hemispheric coherence between frontal-parietal and occipital regions in delta band (T0: 0.20 ± 0.06, T1: 0.30 ± 0.10) was reported. In addition, intrahemispheric theta coherence tended to increase in parietal regions (T0: 0.23 ± 0.08, T1: 0.31 ± 0.07) and to decrease in frontal and occipital regions (T0: 0.25 ± 0.05, T1: 0.22 ± 0.04). Right beta frontal-occipital coherence also tended to decrease (T0: 0.21 ± 0.03, T1: 0.19 ± 0.03) while alpha band tended to increase in parietal regions (T0: 0.22 ± 0.03, T1: 0.29 ± 0.04).

**Figure 3:**
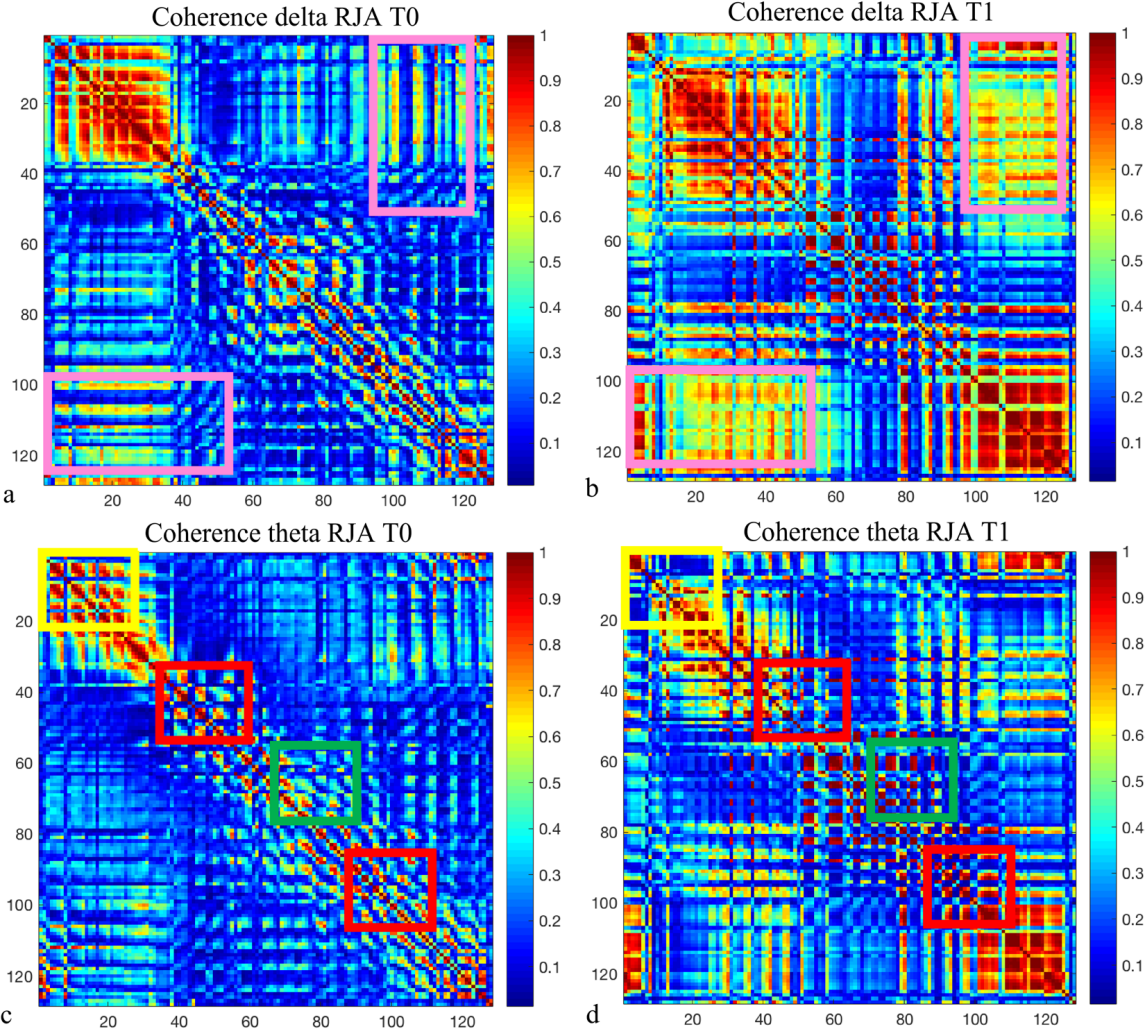
Coherence maps before (**a**,**c**) and after (**b**,**d**) six-months treatment in delta band (**a**,**b**) and in theta band (**c**,**d**) during the responding to joint attention (RJA) task. Maps show an increase of inter-hemispheric frontal-parietal delta coherence (pink boxes) and a reduction of theta band in occipital areas (green boxes), and in frontal areas (yellow boxes) as well as an increase of theta band in parietal regions (red boxes). Coherence data are represented as a matrix in which electrodes are displayed on the x and y axes. Elements of the matrix represent color-coded coherence values between electrodes.

Figure 3 shows the pattern of changes in delta and theta coherence after the six-month treatment in the RJA task.

In the IJA condition, treatment induced a quite different pattern of EEG activity.

As regards power, we observed an increase in frontal gamma activity, specifically in right frontal (T0: 1.71 ± 1.13, T1: 2.66 ± 1.59) and right frontal polar (T0: 1.21 ± 0.21, T1: 1.78 ± 0.39) areas. A concurrent decrease in alpha activity in left frontal area (T0: 7.65 ± 1.95, T1: 4.27 ± 1.9), and in delta activity in left temporal (T0: 7.35 ± 0.1, T1: 6.03 ± 0.1) and left occipital (T0: 6.17 ± 0.51, T1: 5.41 ± 0.25,) areas was also noticed (Fig. 2).

**Figure 4:**
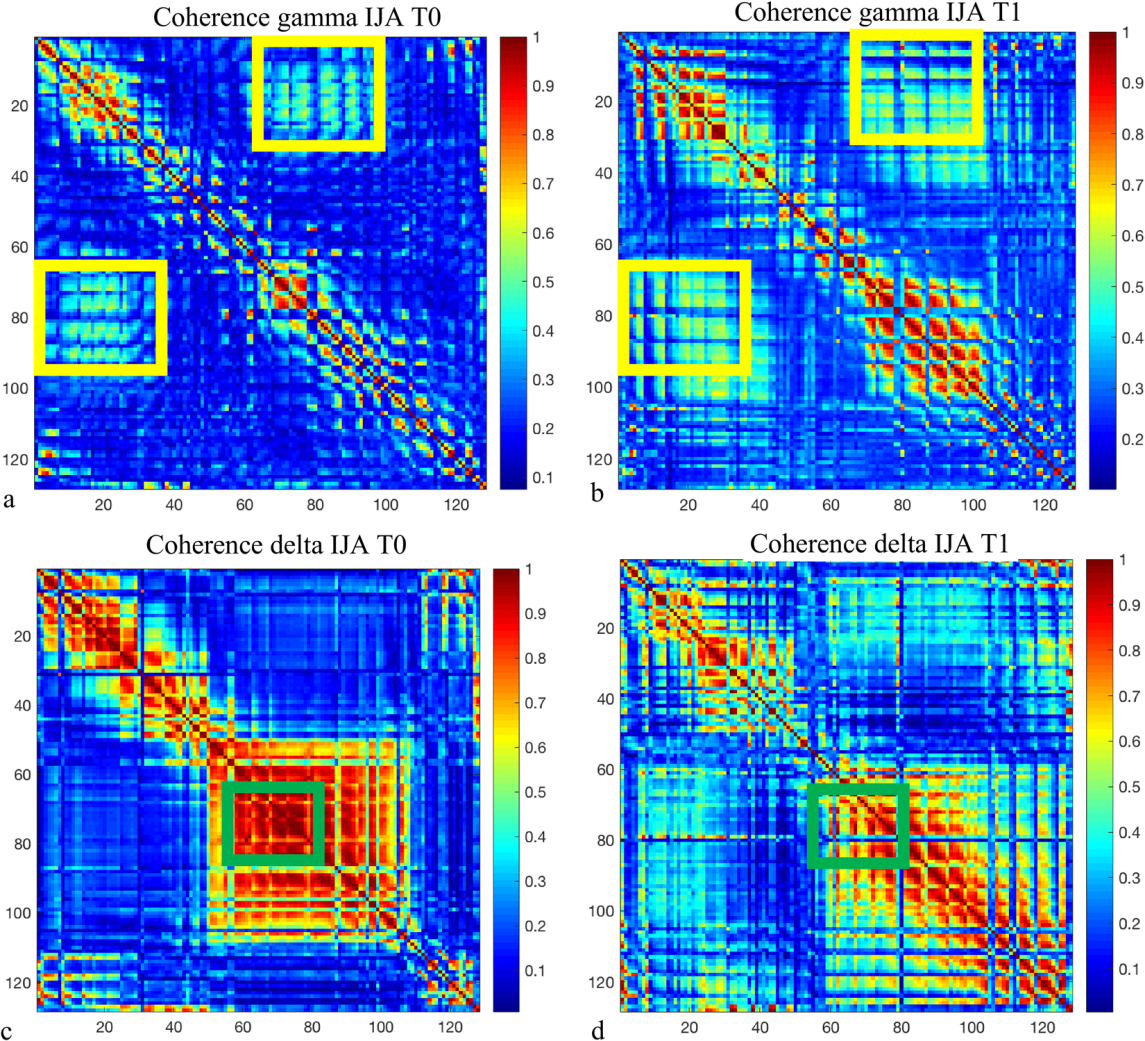
Coherence maps before (**a**,**c**) and after (**b**,**d**) six-months treatment in gamma band (**a**,**b**) and in delta band (**c**, **d**) during the initiating joint attention (IJA) task. Maps show an increase of gamma coherence especially in fronto-occipital connections (yellow boxes) and a reduction of delta band in occipital areas (green boxes). Coherence data are represented as a matrix in which electrodes are displayed on the x and y axes. Elements of the matrix represent color-coded coherence values between electrodes.

As regards coherence we observed a trend of increasing in gamma band in both right (T0: 0.22 ± 0.01, T1: 0.25 ± 0.02) and left (T0: 0.23 ± 0.2, T1: 0.28 ± 0.4) hemisphere coherence between frontal and occipital regions. On the contrary, delta (T0: 0.58 ± 0.2, T1: 0.49 ± 0.1) and alpha (T0: 0.49 ± 0.16, T1: 0.34 ± 0.12) coherence tended to decreased in occipital areas. Figure 4 shows the pattern of increase in gamma coherence and a decrease in delta coherence during the IJA task, after the six-month treatment.

In the control condition, we observed a trend of increasing in intra-hemispheric gamma coherence in temporal regions (T0: 0.20 ± 0.02, T1: 0.25 ± 0.03) and between right occipital and temporal regions (T0: 0.24 ± 0.35, T1: 0.32 ± 0.08). In addition, a trend of reduction in delta occipital coherence (T0: 0.54 ± 0.14, T1: 0.37 ± 0.09), in delta right frontal-occipital coherence (T0: 0.29 ± 0.5, T1: 0.21 ± 0.04) and in alpha left fronto-temporal coherence (T0: 0.24 ± 0.35, T1: 0.32 ± 0.08) was noticed.

### Correlations between EEG and eye-tracking measures at T1

**Figure 5:**
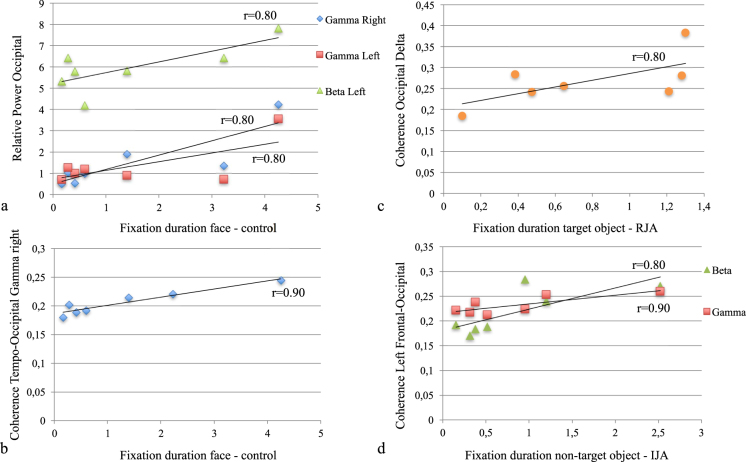
Correlations between cumulative fixation durations and electrophysiological measures. (**a**) Positive correlation between gamma and beta activity in occipital regions and cumulative fixation duration for face in control condition; (**b**) positive correlation between cumulative fixation duration for face and right temporal-occipital coherence in gamma band in control condition; (**c**) positive correlation between intra-hemispheric coherence in delta band and cumulative fixation duration for target object in responding to joint attention (RJA) task; (**d**) positive correlation between left frontal-occipital coherence in gamma and beta bands and cumulative fixation duration for non-target object in the initiating joint attention (IJA) task.

Correlations between eye-tracking measures and EEG features at T1 were explored, to investigate the neurophysiological underpinnings of gaze processing. Cumulative fixation duration for face was positively correlated with relative gamma and beta power in occipital in the RJA (r = 0.67) and the IJA tasks (r = 0.69). In the control condition, a positive correlation was observed between cumulative fixation duration for face and gamma and beta activity in occipital areas (r = 0.80) (Fig. 5a), and between cumulative fixation duration for face and the right temporal-occipital coherence in gamma band (r = 0.90) (Fig. 5b).

In the RJA task, a trend of positive correlation between the inter-hemispheric occipital delta coherence and the cumulative fixation duration for target object was noticed (r = 0.80) (Fig. 5c). Finally, in the IJA task, a positive correlation between coherence between frontal and occipital areas in beta (r = 0.80) and gamma bands (r = 0.90) and cumulative fixation duration for non-target object was observed (Fig. 5d), with both features increased after the treatment.

## Discussion

In this study, we used an integrated approach to assess the neurophysiological circuitry during joint attention tasks and their changes after treatment in children with ASD.

Our results suggest that: (1) initiating and responding JA subtend with both overlapping and specialized neural circuitries, elicited by the two different tasks; (2) trends of changes in brain activity and connectivity are observed after treatment; (3) these modifications subtend changes as measured by eye-tracking.

Literature proved that IJA and RJA reflect overlapping but also different types of social information processing networks that rely on both shared and unique neural circuitries^25^. In particular, according to previous EEG and imaging studies in infants, the IJA competence seems to involve, during early development, mainly the frontal regions^26^,^27^, while the RJA is more related to parietal and temporal cortical processes^27^,^28^. However, it is also plausible that, although IJA and RJA follow distinct neural pathways during development, they later integrate in a frontal-posterior pattern of connectivity. Indeed, a recent combined fMRI-eye-tracking study in typically developing adults has identified a common frontotemporal-parietal network for RJA and IJA task^29^. The comparison of the neural circuitries before treatment, in our sample of ASD subjects, confirms such integration, although with some differences between the two tasks.

As regards the overlapping regions, in both IJA and RJA tasks, we observed an involvement of frontal regions and temporal brain areas. Frontal regions have been implicated in several tasks related to joint attention including reward^15^, theory of mind^30^ executive functions^31^ and observing goal-directed actions^32^. The frontal activation in joint attention is consistent with other studies on typically developing children^27^, and may be related to functions like working memory and related dual-task processing, as well as to the representational encoding of visual stimuli related to the task. In addition, the fact that the joint attention skill has been linked to language development^33^, and that temporal processes are involved in word discrimination and comprehension, suggest the existence of possible links between the activation of temporal circuitries and JA function^2^.

In particular, it has been hypothesised a key role for the STS, being activated both during language processing and during the processing of biological motion and social attention, such as eye gaze processing, in mediating language and attention functional domains. This mechanism could occur by integrating changing auditory and visual cues and extracting social or communicative significance of these cues, which may be critical to word learning. The STS’ role in interpreting social and speech input, could explain why impairments in this region may underlie many of the social and language abnormalities observed in ASD individuals^34^.

Concerning circuitry specialisation, we observed, in RJA task, an involvement of the parietal regions, as shown by both power and coherence analyses, which was not present in IJA task. The reported function of the parietal lobe consisting of enabling shifts of attention by disengaging attention from its current focus^35^ may be employed when a child redirects his/her attention in response to head and gaze shift of the experimenter during the RJA trial. Conversely, in the IJA task there was an involvement of the occipital regions in both power and coherence and in the connectivity between occipital and frontal regions, which was not present in the RJA task. It has been suggested that the double activation of both frontal and posterior regions in IJA could be explained with the posterior-to-anterior model of development^27^. The posterior component of this system is thought to develop, indeed, before the anterior component. Between 3 and 6 months of age, infants become able to shift attention by inhibiting or disengaging attention from an immediate focus. The development of this ability is thought to reflect a posterior “orienting” attention system regulated, in part, by components of the parietal cortex as well as associated midbrain and thalamic areas^36^.

Previous fMRI studies in children and adults with typical development suggested that frontal and temporal areas are involved with both RJA and IJA tasks^29,37^, while the parietal region is mainly associated with the RJA task^29^,^38^. The present results suggest that the involvement of brain regions in our sample of high-functioning children with ASD resembles that of typically developing individuals as far as JA circuitries are concerned.

Notably, before treatment, a different involvement of EEG bands between the two JA tasks was also observed. In RJA task, there was an increase of theta and alpha, while delta band increased in IJA. Consistently, theta and alpha power have been related to eye gaze processing^39^. In particular theta oscillations may imply the activity of the frontal cortex including an attention network involved in executive and voluntary control of attention^40^, while alpha has been related, in adults, to attention mechanisms that actively suppress distracting information to focus on the relevant input^41^. Conversely, delta band has been linked to learning, motivation and reward processes^42^.

After treatment, some trends of changes were observed both in terms of power and coherence in RJA and IJA circuitries. In particular, in RJA task power decreased both in frontal and temporal regions, while coherence decreased between frontal and occipital areas and increased between frontal and parietal areas, as well as within parietal regions. Thus, it seems that after treatment for RJA there is a strengthening of the fronto-parietal network reducing the activation of the other circuitries. Such changes are mainly concerned with theta and alpha bands, which are also involved in gaze perception, as observed before treatment. Jaime and colleagues^24^ found a significant decrease in alpha coherence during the RJA task in children with ASD compared to TD and they also found a significant positive correlation between alpha coherence and the “Ready the Mind in the Eyes Test” in TD, suggesting that this measure is a positive biomarker for social functioning. We also observed a fronto-parietal increase of coherence in delta band after treatment. EEG delta activity increases mainly in frontal areas in different tasks involving semantic and working memory skills^43^. Moreover, the increase in frontal delta during cognitive tasks has been linked to the perceptual switching of objects^44^. These essential functions of the brain are likely to play a crucial role in the RJA task, thus possibly explaining the increase of delta band in this condition.

In IJA we observed an increased activation of frontal regions and an increased coherence between frontal and occipital areas. Conversely, coherence decreased within occipital regions, while power decreased in temporal and occipital regions. We could speculate that the treatment might strengthen some frontal abilities that are needed to achieve efficient IJA, i.e. the perception of a social partner^45^, or the ability of making judgments about others and oneself^46^, and of inferring others’ mental states^47^. In addition, after treatment the connectivity between frontal and occipital areas increased. This anterior-posterior connectivity could ameliorate the communication between visual information and cognitive control according to the view “where my eye’s go, my behaviours follows”^2^. These changes were particularly evident in the gamma band, which has an important role in sensory integration and cognitive processing^48^, as well as in sustained attention^49^. The increase of gamma activity after treatment might be therefore an index of an improvement of the abilities that serve to the child to effectively achieve the JA goal.

The 6-month treatment was accompanied by changes in eye-tracking measures, which partially correlated with the EEG features. In RJA, we observed a tendency to increase the gaze duration to target object, suggesting an increased ability to follow the gaze of the actress and to engage with the attended object^4^. The correlation of delta coherence and cumulative fixation duration for target object after treatment may suggest that the process of gaze following is sustained by an increase in delta coherence. This result agrees with the assumption for the role of frontal delta in working memory and object perception^43,^ ^44^. Interestingly, delta oscillations are also associated with motivational processes, and with the research of internal, motivationally salient, cues that signal potential rewards^50^,^51^. It is possible that these latter aspects might be the actual targets of the therapy, and that they might exert an indirect, beneficial effect, on the child ability to respond to JA.

In IJA, a tendency to increase cumulative fixation duration for the non-target object was also observed. Although this result may seem counterintuitive, our previous findings on toddlers with TD and ASD children^19^ showed that, in this type of IJA task, the TD children tended not only to look at the moving (target) object, but also to divide their attention between the moving and the other object. Conversely, ASD children tended to focus their attention only on the moving object. A possible explanation of this behaviour is that TD subjects foresee a possible movement of the still non-target object, while this capacity is impaired in ASD patients. In fact, difficulties in anticipation have been described as one of the strongest indicators of ASD^52^ and as a sign of mirror neuron system dysfunction^53^. The increased attention to the non-target object after the treatment protocol seems to suggest that children likely increased the abilities to anticipate object movement, more similarly to TD people. Such changes were also correlated with an increased gamma activity in frontal areas, which has been also linked to anticipation of visual stimuli skills^54^.

Eye-tracking measures also showed an increased attention for face in all the conditions. Looking at social stimuli, such as face, is a prerequisite for joint attention behaviour^4^, therefore likely representing a positive treatment effect on children involved in the study. In the control condition, where only the face was present, we observed an increase in beta occipital activity, which is known to be mainly associated to the processing of emotional face stimuli^55^, suggesting an increased ability of processing the social stimuli, which is confirmed by the positive correlations between cumulative fixation duration for face in control condition, and occipital beta, together with gamma.

Due to the small sample size, we only reported the effect sizes of these correlations (Pearson’s r) and we obtained quite large effects (0.67 < r < 0.90).

Despite the novel approach presented in this study and the promising results of the proposed method in characterising brain and visual patterns linked to joint attention in ASD, some important limitations need to be considered when interpreting these results. A first limitation is represented by the small sample size; in particular, only a subgroup of seven subject was longitudinally assessed and thus we could only report qualitative trends and we could not drive solid conclusions about the treatment effect. The significance of results at T0 in the whole group were quite strong (between 0.01 and 0.003) and mostly survived the stringent Bonferroni correction. Moreover, we also relied on effect sizes, which are not affected by sample size, and we obtained large effect size values, suggesting the reliability of the results we achieved.

The study was designed as a pilot research, aiming at testing the feasibility of using a novel protocol, based on the integration of high-density EEG and eye-tracking, to assess the effect of a 6-month intensive treatment on the neurophysiological and visual correlates of joint attention. Thus, only a small number of patients was recruited, with the aim of having a homogeneous group of high-functioning male children with ASD, who could complete the assessment protocol and the treatment, while limiting the overall dropout. The findings of the study need to be replicated with a larger sample to verify the results of this pilot investigation and to prove the efficacy of the treatment.

Another limitation is represented by the absence of a control group of ASD patients, which did not perform the treatment. In fact, the study design involved a within-group and pre-post treatment comparison, in which each child was a control for himself. Future studies might also add a control group of children with ASD undergoing a treatment “as usual”, in order to make us more confident that the longitudinal changes, eventually observed, were effectively due to the treatment. In addition, we could also include a sample of typically developing children to understand how the brain activation and eye-tracking response is different to that of children with ASD in this specific responding to joint attention and initiating joint attention tasks.

It is of note that in the current study we restricted eligibility to male patients, in order to control for potential gender-related heterogeneity. Thus, the lack of females within the ASD sample prevents us from identifying what their pattern of EEG/eye-tracking change would be. Recent literature that specifically explored the JA skills in children with ASD suggested no gender differences between girls and boys evaluated through a structured session of play interaction^56^, or a parent report scale focused on JA^57^. We can therefore speculate that females with ASD, carefully matched on developmental and diagnostic variables with the males recruited in this study, would have the same pattern of EEG/eye-tracking response of JA. An ad hoc investigation of sex differences in the tasks proposed in the current study should be planned in order to verify this hypothesis.

## Conclusions

Overall, these findings suggest the existence, in high-functioning children with ASD, of neural circuitries responsible of joint attention process, with some regions of overlap between the two components and some regions of specialization, as previously observed in typically developing subjects^25^ and in adults with ASD^22^. In addition, the results of this study may suggest that the treatment affects neurophysiological and eye-tracking measures. The intensive rehabilitative intervention implemented in the MICHELANGELO project, mainly focused on joint attention and imitation, has likely changed the ability of children to process joint attention stimuli, both at a behavioural and at a neural circuitry level. The behaviour-brain link is supported by the integrated acquisition and analysis of eye-tracking and EEG measures. These results could go in the directions of previous studies applying neuroimaging methods demonstrating brain plasticity caused by behavioural treatment in ASD, which result in a brain pattern more similar to those of subjects with typical development^58^. Thus, repeated neuroimaging evaluation might represent a promising tool for the investigation of neural changes in response to treatment in patients with ASD. The research was designed as a pilot study, to test the feasibility of this new methodology, thus considerably limited in the sample size. Further data collections, and larger samples, are needed to confirm these preliminary results, the possibility to accurately characterise the neurophysiological patterns linked to different behaviours, and to longitudinally monitor the effect of the treatment through objective measurements of the child’s response. Importantly, a control group will be necessary in future steps of the study, in order to compare the results obtained in children with ASD and to further ensure that the modifications observed in the ASD sample would be actually due to treatment intervention.

## Method

### Participants

A total of 14 high-functioning children with ASD were recruited from the IRCCS Stella Maris Foundation. Two of them did not complete the protocol, while one was not included in the analyses because of missing EEG data due to technical problems during the acquisition. Therefore, the final sample consisted of 11 children with ASD (all males, age range = 6.1–9.2 yrs, mean age = 7.5 ± 1.1 yrs) (Table 1). A subgroup of 7 of these children was longitudinally assessed after 6 months of treatment. The ASD diagnosis was formulated according to the DSM-5 criteria^59^ and confirmed by the Autism Diagnostic Observation Schedule-2 (ADOS-2)^60^ and the Autism Diagnostic Interview-Revised (ADI-R)^61^. The adaptive behaviour was assessed using the Vineland Adaptive Behaviour Scale-II (VABS-II)^62^. A multidisciplinary team - including a senior child psychiatrist, and two clinical child psychologists experienced in ASD - conducted the diagnostic assessment during a 5-day extensive evaluation. The Wechsler Intelligence Scales for Children-fourth edition (WISC-IV)^63^ were used to assess the Full Scale Intelligence Quotient (FSIQ). The study was approved by the IRCCS Stella Maris Foundation’s Ethical Committee and all the parents signed a written informed consent form to participate. The participants were treated in accordance with the declaration of Helsinki.

**Table 1:** Participants characteristics.

Subjects	Age *(in years)*	ADOS-2 *CS*	ADI *QARSI*	ADI *QAC*	ADI *RRSPB*	ADI *ADE 36 months*	WISC-IV *FSIQ*	Vineland-2 *ABC*
S1*	6.1	3	10	8	3	3	117	75
S2*	6.3	3	12	10	4	3	84	76
S3*	7.3	3	14	12	3	1	97	85
S4*	7.8	4	16	11	3	5	98	75
S5*	6.8	4	10	8	3	1	92	73
S6*	8.10	5	12	9	3	3	127	75
S7*	9.2	5	11	9	3	1	111	74
*S8*	7.6	6	22	12	2	2	123	78
*S9*	8.3	4	9	8	1	1	117	77
*S10*	9.1	8	21	18	11	3	100	94
*S11*	6.1	5	12	11	4	2	118	97

*CS = Comparison Score; QARSI = Qualitative Abnormalities in Reciprocal Social Interactions; QAC = Qualitative Abnormalities in Communication; RRSB = Restricted, Repetitive, and Stereotyped Patterns of Behavior; ADE = Attività Didattica Elettiva (Elective Teaching Activities); FSIQ = Full Scale – IQ; ABC = Adaptive Behaviour Composite*.

*Subjects who performed the 6-month treatment.

### Intervention

Participants performed one 40-min treatment session each week for six months, during which a technological platform was used to record neurophysiological, autonomic and behavioural data^64^,^65^. The first half of the session consisted of a therapeutic protocol with gradually increasing difficulty based on imitation (IM), and on IJA and RJA in the context of a play-based setting. In the IM tasks, the therapist asked the child to imitate what he/she was doing. In an example of JA task, a therapist red a social story while a second therapist dramatized some parts of the story: in the IJA task the child was expected to spontaneously look at the second therapist, and then shift the gaze toward the first therapist in order to share the event; in the RJA task, the first therapist looked at the child and then vocalised, pointed or looked at the second therapist in order to elicit a JA response in the child. During the second half of the session the therapist proposed the children some socio-cognitive tasks supported by the use of the serious game “GOLIAH”, developed within the MICHELANGELO project^66,67^, that maps IM and JA stimuli derived from the Early Start Denver Model^68^. Importantly, the games were implemented on two tablets, one managed by the therapist and the other by the child, in order to drive them to cooperate to achieve the goal of the game. In addition to the treatment sessions with the therapist at the hospital, the training with “GOLIAH” also included five 20-minute sessions per week with parents playing with their children at home.

### Data acquisition

EEG and eye-tracking data were concurrently acquired in all ASD children at the beginning (T0) and, for a subgroup of 7 children, at the end (T1) of a six-month treatment. The EEG system and the eye-tracker were synchronized using a switch to simultaneously send the stimuli from the stimulus PC to the Net Station and the eye-tracker PC. EEG data were continuously recorded with a 128 channels HydroCel Geodesic Sensor Net (HCGSN 128; Electrical Geodesics Inc). Data were acquired at a sampling rate of 500 Hz, setting impedances below 50 kΩ, and using a band-pass filter between 0.1 and 100 Hz, and a notch filter at 50 Hz for a visualization purpose. Children’s gaze was recorded by the SMI Eye Tracking (SensoMotoric Instruments, Germany), with a sample rate of 500 Hz and an accuracy of more than 1 degree of visual angle. The eye-tracker was placed in front of the subject (approximately 50 cm), just below a 22-inch flat screen monitor where the stimuli were presented through the SMI Experiment Center Software^TM^.

### Stimuli

**Figure 6:**
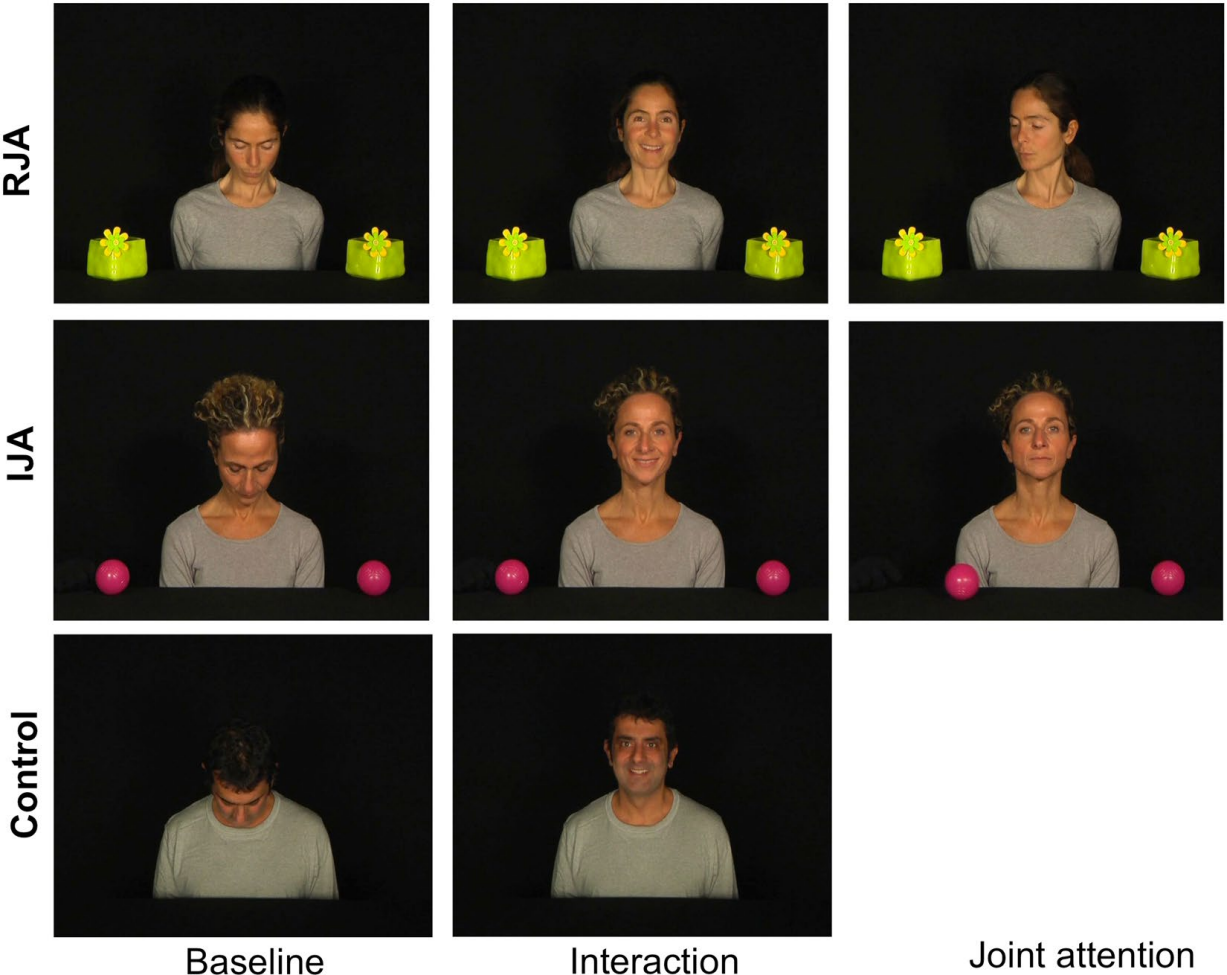
Screen shots of the videos of the different tasks. (**a**) Responding to joint attention task (RJA), (**b**) initiating joint attention task (IJA) and (**c**) control task. The RJA and IJA tasks are divided into three different phases: looking down, interactive, joint attention. The control task is divided in two phases: looking down and interactive.

The JA paradigm was created using the E-Prime software. The experiment began with a 5-point calibration routine. Stimuli consisted in video sequences, with an actor/actress visible in the foreground on black neutral background with two identical toys placed on the floor in front. The experimental proof of the task involves three conditions: the RJA, the IJA and a control condition without objects (Fig. 6).

**Figure 7:**
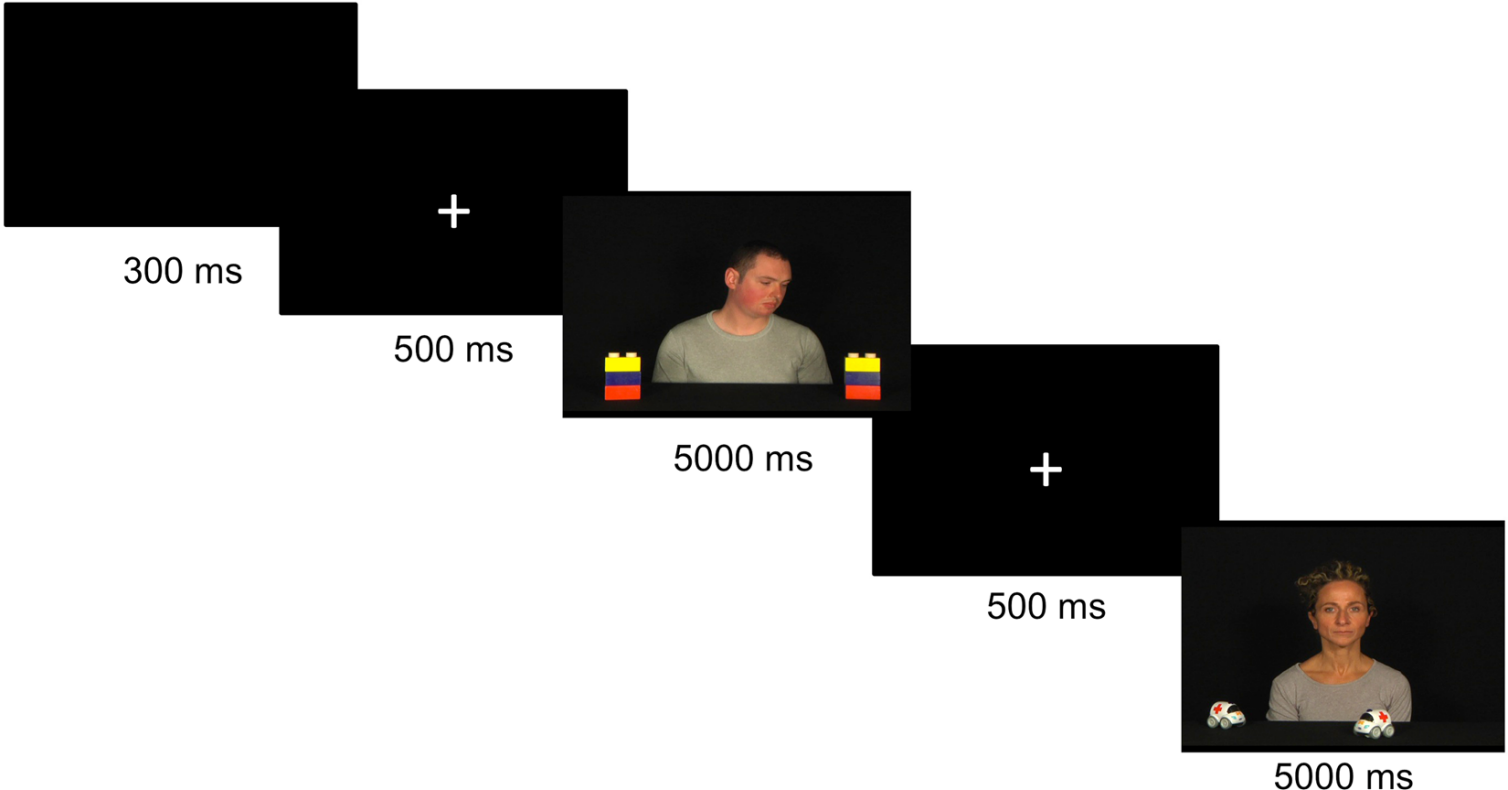
Experimental paradigm.

In the RJA, after the initial “looking down” in which the actor/actress looks down (500 ms), he/she smiles to the child in order to invoke the focus (“interactive” phase, 2000 ms). Following this interactive stage, the actor/actress turns his/her head and eyes to one of the objects, realising the “joint attention” phase (2500 ms). In the IJA, the first two phases are identical to the RJA tasks. In the third phase of the IJA task while the actor/actress maintains direct gaze towards the camera and assumes a neutral facial expression, one of the two objects placed on the floor moved unexpectedly (“joint attention” phase, 2500 ms). In the Control condition, after the initial 500 ms “looking down” phase in which the actor/actress looks down, he/she smiles to the child in order to invoke the focus (“interactive” phase, 4500 ms). Each video clip of 5 seconds was interspersed with a fixation cross (500 ms) to control the attention of the child (Fig. 7). Two blocks of 40 repetitions for each condition were presented to each child. Stimuli were presented in a randomised order.

### Eye-tracking data analysis

Considering the focus of the paper and the JA construct, the measures were computed only for the JA segment of the RJA and IJA. Measures were computed by extracting raw data and analysing them in Matlab (MathWorks, Natick, MA, USA), using homemade scripts. The following areas of interests (AOIs) were selected: model’s face, target object, non-target object. To explore the child’s engagement with each AOI, cumulative fixation duration within the selected AOI, which is defined as the time spent on that AOI, was analysed. A fixation threshold of 60 ms was applied to avoid unconscious looking. Cumulative fixation duration on a specific AOI was computed as a percentage of the total, which means cumulative fixation duration on that AOI relative to the participants’ on-trial cumulative fixation duration. In the Control condition, we only computed cumulative fixation duration for the face during the interaction phase.

### EEG data analysis

Data were pre-processed using EEGLAB Toolbox^69^. Data were band-pass 0.5-70 Hz to remove slow linear trends and high frequency noise and a notch filter (45–55 Hz) was applied to remove power line interference. Ocular and muscular artefacts were first removed by visual inspection. In particular blink artefacts were identified as segments of data having deflection > 150 μV, while ocular flutters, or muscle movement artefacts as segments having deflections of 50 μV relative to baseline. Channels where the amplitude exceeded 200 μV, or that had zero variance, for at least 20% of the recording, were removed from the analysis, and subsequently interpolated. Data were re-referenced to the average of all channels. Data were then epoched according to the event markers for all the conditions considering a pre-stimulus of 800 ms, and their termination coinciding with the end of the video.

For removing the residual artefacts on epochs due to eye movements or muscles the *fastica* algorithm of EEGLAB was applied, then the algorithm ADJUST^70^ was used for the selection of the components to be removed. Once major artefacts were removed, we rechecked the epochs for residual artefacts using the EEGLAB’s epoch rejection tools. In addition, we excluded the EEG epochs in which the child looked at the screen for less than 200 ms, as measured using the eye-tracking. Data were baseline corrected considering the pre-stimulus segment of the video conditions as control signal.

Pre-processed data were imported in a home-made Matlab tool for quantitative EEG analysis. Quantitative features were extracted from each epoch, and then averaged over all epochs, separately for each of the three conditions. First, the Power Spectral Density was calculated using the Welch method (Hanning window of 2 s, 50% overlap). From the Power Spectral Density, the relative power for each of the main EEG frequency bands was obtained: delta (1 – 4 Hz), theta (4–7 Hz), alpha (8–13 Hz), beta (14–29 Hz) and gamma (30–80 Hz). Coherence at each frequency band was then calculated for each pair of electrodes as a measure of phase locking.

A reduction of data was applied for comparing features: the power of the 128 electrodes was averaged across specific brain areas and coherence measures calculated between these areas. According to previous studies, the following areas were defined: left frontal polar, right frontal polar, left frontal, right frontal, left temporal, right temporal, left parietal, right parietal, left occipital and right occipital.

### Statistical analysis

Statistical analysis was performed using SPSS 20.0 for Mac only at T0 in the whole sample. The descriptive statistics for each of the dependent variables were examined. With the aim to explore the neural correlates of RJA and IJA, we performed a repeated-measures MANOVA on EEG measures at T0, in which the task (RJA or IJA) was the within-subject variable. Relative powers and coherences within brain regions were explored as independent variables. When powers and coherences where tested, band was also added as a within-subject factor. Age was used as covariate for all the analyses. Since the MANOVA only tells if there is an overall difference between the variables (i.e. coherence values), but it does not tell which specific variable (i.e. coherence in temporal regions in delta band) differ, univariate tests were performed when an overall statistically significant difference was observed. Univariate analyses were conducted using paired sample t-tests comparing variables for each band. When performing univariate tests, Bonferroni correction was applied resulting in a significance of p < 0.01 (5 bands). Effect sizes (by partial eta squared-η^2^ for MANOVA and Cohen’s d for t-tests) were reported as they take into account mean differences and standard deviations without being sensitive to sample sizes. According to the literature, for η^2^ values between 0.01 and 0.06 are generally considered a small effect, between 0.06 and 0.14 a medium effect and those above 0.14 are regarded as a large effect; while d = 0.2 be considered a small effect size, 0.5 represents a medium effect size and 0.8 a large effect size^71^.

Because of the small size of the sample, which was longitudinally assessed, and the exploratory nature of our study, we did not report quantitative results for the longitudinal eye-tracking and EEG data but instead we only reported qualitative trends. To examine the relationship between eye-tracking and EEG measures effects sizes (Pearson’s r), rather than statistical significance, were used to interpret meaningful correlations.
